# Clinical characteristics of patients with chronic cough in Guangdong, China: a multicenter descriptive study

**DOI:** 10.1186/s12890-021-01642-z

**Published:** 2021-09-27

**Authors:** Kefang Lai, Lianrong Huang, Haijin Zhao, Feng Wu, Guocui Zhen, Haiyan Deng, Wei Luo, Wen Peng, Mei Jiang, Fang Yi, Jianxin Sun, Pusheng Xu, Yuqi Zhou, Yinji Xu, Xiaoling Yuan, Yiju Zhao, Meihua Chen, Yong Jiang

**Affiliations:** 1 State Key Laboratory of Respiratory Disease, National Clinical Research Center for Respiratory Disease, Guangzhou Institute of Respiratory Health, The First Affiliated Hospital of Guangzhou Medical University, Guangzhou, Guangdong People’s Republic of China; 2 Chronic Airway Disease Laboratory, Department of Respiratory and Critical Care Medicine, Nanfang Hospital, Southern Medical University, Guangzhou, Guangdong People’s Republic of China; 3 Huizhou Third People’s Hospital, Guangzhou Medical University, Huizhou, Guangdong People’s Republic of China; 4 The First People’s Hospital of Foshan, Foshan, Guangdong People’s Republic of China; 5 Shenzhen Second People’s Hospital, Shenzhen, Guangdong People’s Republic of China; 6 The Second People’s Hospital of Zhaoqing, Zhaoqing, Guangdong People’s Republic of China; 7 The Second Affiliated Hospital of Guangzhou Medical University, Guangzhou, Guangdong People’s Republic of China; 8 The Third Affiliated Hospital of Sun Yat-sen University, Guangzhou, Guangdong People’s Republic of China; 9 The Second Clinical College of Guangzhou University of Chinese Medicine, Guangdong Provincial Hospital of Chinese Medicine, Guangzhou, Guangdong People’s Republic of China; 10 Zhongshan Hospital of Sun Yat-sen University, Zhongshan People’s Hospital, Zhongshan, Guangdong People’s Republic of China; 11 The Fifth People’s Hospital of Dongguan, Dongguan, Guangdong People’s Republic of China; 12 The Third People’s Hospital of Dongguan, Dongguan, Guangdong People’s Republic of China; 13 Shenzhen Hospital of Integrated Traditional Chinese and Western Medicine, Shenzhen, Guangdong People’s Republic of China

**Keywords:** Chronic cough, Clinical characteristics, Demographics, China

## Abstract

**Background:**

The clinical characteristics of patients with chronic cough are reported only in single-center survey in China, being significantly different from that in western countries. Here, we performed a multicenter study to describe the clinical characteristics of chronic cough patients.

**Methods:**

A cross-sectional observational survey was conducted in thirteen tertiary hospitals of Guangdong, South China. Relevant data were recorded using a standardized questionnaire and analyzed, including demographics, educational attainment, cough features, and concomitant symptoms.

**Results:**

Of 933 patients in this study, the median age was 40.0 (IQR 31.0–52.0) years with a peaked age of 30–39 years. The proportion of females (487, 52.2 %) was comparable to that of males (446, 47.8 %). Up to 81.9 % of the patients were non-smokers. More than two-thirds of the subjects with chronic cough had a low educational level. The median cough duration was 6.0 (IQR 3.0–24.0) months, and 73.0 % of chronic cough patients presented with dry cough. Laryngeal paresthesia was the most common concomitant symptom (704, 75.5 %), followed by rhinitis/sinusitis-related (350, 37.5 %) and respiratory symptoms (322, 34.5 %). Rhinitis/sinusitis-related symptoms more frequently occurred in patients with productive cough than in those with dry cough (49.0 % vs. 33.0 %, *P* < 0.001). Moreover, female patients displayed an older age and a higher prevalence of nocturnal cough compared to male patients (both *P* < 0.05).

**Conclusions:**

Our results show an equal gender, young profile and laryngeal paresthesia in patients with chronic cough, and different clinical features between females and males.

## Background

Chronic cough is a common complaint for patients seeking medical attention with an estimate prevalence of 10 % around the world, particularly in Oceania, Europe and the United States [1]. Many patients suffered a marked decrement in quality of life and enormous economic burden due to chronic cough [2, 3]. Growing evidence suggested that the common medical conditions associated with chronic cough were cough variant asthma (CVA), upper airway cough syndrome (UACS), nonasthmatic eosinophilic bronchitis (NAEB) and gastroesophageal reflux cough (GERC) [4–6]. A worldwide survey reported that two-thirds of chronic cough patients were females with a peaked age of 60–69 years across western countries [7]. Whilst most single-center studies in China showed that chronic cough patients displayed a middle-aged predominance with equal gender distribution [8, 9], being different from those in western countries. These age and gender disparities, however, should be further investigated among the cough population living in varied regions.

An increased sensitivity to the tussigenic agents such as capsaicin, citric acid, and ATP was found in patients with chronic cough in comparison with healthy volunteers [10, 11]. Besides, chronic cough was also associated to occupational exposure, cigarette smoking, unhealthy lifestyle as well as ambient air pollution [7, 12, 13]. The Pearl River Delta, located in Guangdong, is a highly rapid industrialization and urbanization region with pollution intensive industries. Up till now, the clinical characteristics of chronic cough patients were reported in single center survey in Guangzhou, the capital of Guangdong province [8, 9]. Thus, the multicenter data is still lacking in China. However, investigations on the clinical features of chronic cough will be crucial to improve efficacious treatment and management strategies. Therefore, we performed a multicenter observational study to describe the demographics and clinical characteristics of chronic cough patients in Guangdong, China.

## Methods

### Study design and participants

This was a cross-sectional study conducted in respiratory specialist clinics of thirteen tertiary hospitals from seven cities of Guangdong between August 2017 and August 2018. The inclusion criteria included cough as the predominant or sole symptom lasting more than 8 weeks, no overt abnormality of chest imaging, and age ≥ 15 years old. We excluded the patients with obvious dyspnea or wheeze, concomitant severe systemic diseases, or lacking of independent ability of filling out the questionnaire. All participants completed a standardized questionnaire via a face-to-face survey. Relevant information was extracted and analyzed, including demographics, educational attainment, duration, characteristics, timing, seasonality, concomitant symptoms, smoking status, as well as exposure history. Concomitant symptoms consisted of pharyngeal paresthesia, rhinitis/sinusitis-related, reflux, as well as respiratory symptoms (wheezing, dyspnea, chest tightness, etc.). This study was approved by the Ethics Committee of the First Affiliated Hospital of Guangzhou Medical University (IRB No.201,778). All participants provided the written informed consents prior to study.

### Statistical analysis

All statistical analyses were performed using SPSS statistical software version 22.0 (SPSS Inc., Chicago, IL, USA). Age and cough duration were expressed as the median (interquartile range, IQR) and were compared using Mann-Whitney U test. The remaining parameters (e.g., gender, educational level, characteristics, timing, seasonality, associated symptoms, smoking status and exposure history) were presented as n/N (%), and the comparisons between groups were examined by Chi-square test or Fisher’s exact test. *P *value < 0.05 was considered as statistically significant.

## Results

### Characteristics of the study population

**Fig. 1:**
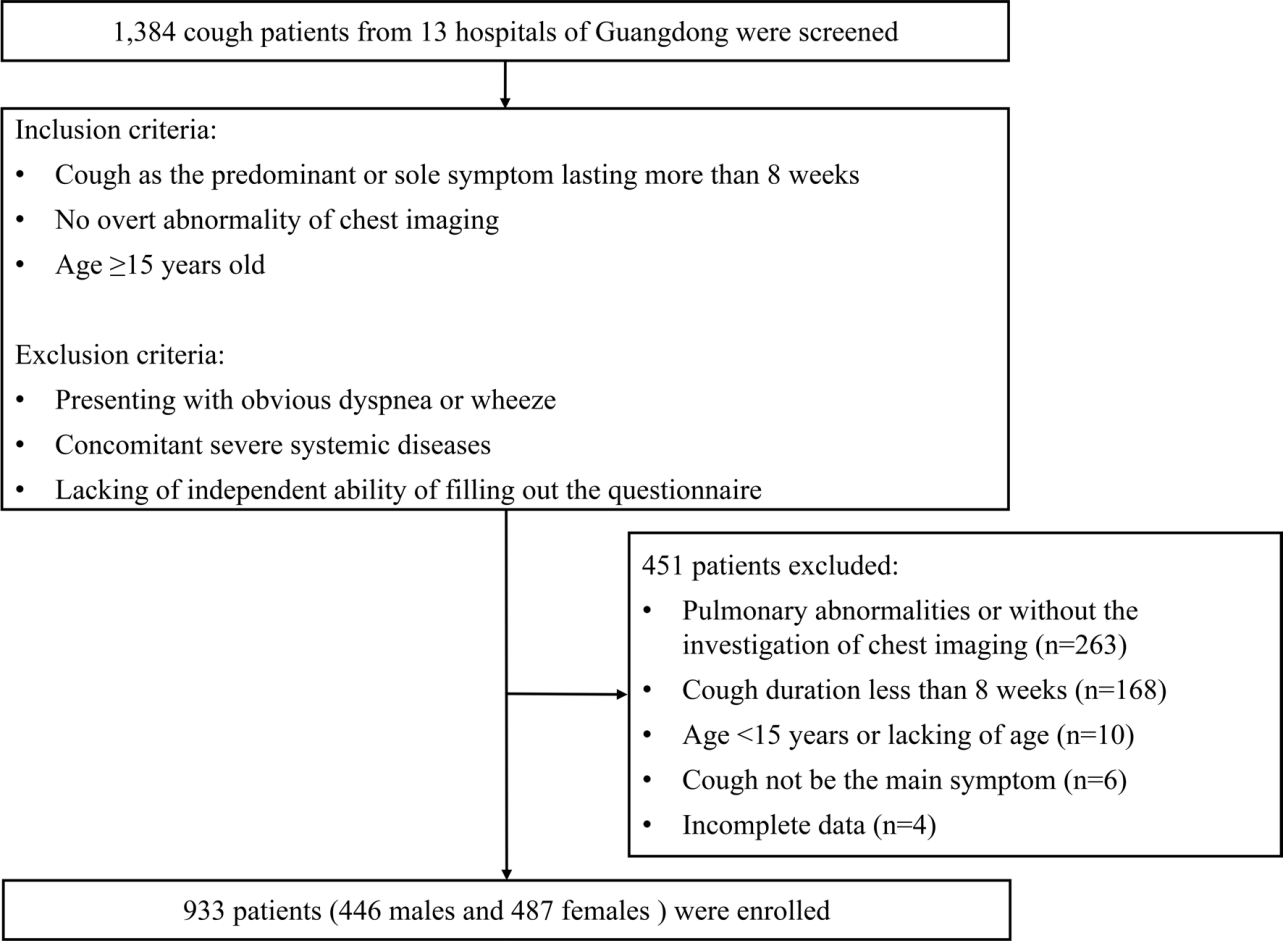
Flow diagram of patients screening

**Fig. 2:**
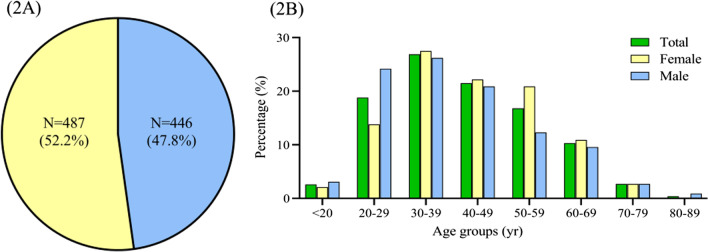
Gender and age distribution of overall chronic cough patients presenting to the clinics. **A** Gender distribution of all chronic cough patients. **B** Age distribution of all chronic cough patients. Total-percentage (%): ratio of the number of patients in different age to the total number of cough patients (n = 933). Female/male-percentage (%): ratio of the number of patients in different age to the number of females (n = 487) or males (n = 446)

A total of 1,384 subjects were consecutively screened and 933 of them were enrolled in this study (Fig. 1). The baseline clinical features of the study population were shown in Table 1. The median age was 40.0 (IQR 31.0–52.0) years and 52.2 % were females (Fig. 2A). Patients aged 20–59 years accounted for 84.0 %, and the most common age of presentation was 30–39 years (251, 26.9 %), followed by 40–49 years (201, 21.5 %), 20–29 years (175, 18.8 %) (Fig. 2B). No matter in males or in females, the prevalence of chronic cough in different age group exhibited a roughly similar distribution as the whole study population. However, slight differences of the peak age range appeared in between females and males. In other word, the peaked age range was 30–59 years in women while that was 20–49 years in men.

**Table 1:** Clinical characteristics of patients with chronic cough between male and female groups

Features	All	Male	Female	P-value
*No.*	933 (100)	446 (47.8)	487 (52.2)	–
*Age, years*	40.0 (31.0–52.0)	37.0 (29.0–50.0)	43.0 (33.0–53.0)	< 0.001
< 20 years	24/933 (2.6)	14/446(3.1)	10/487 (2.1)	0.295
20–29 years	175/933 (18.8)	108/446 (24.2)	67/487 (13.8)	< 0.001
30–39 years	251/933 (26.9)	117/446 (26.2)	134/487 (27.5)	0.659
40–49 years	201/933 (21.5)	93/446 (20.9)	108/487 (22.2)	0.623
50–59 years	157/933 (16.8)	55/446 (12.3)	102/487 (20.9)	< 0.001
60–69 years	96/933 (10.3)	43/446 (9.6)	53/487 (10.9)	0.533
70–79 years	25/933 (2.7)	12/446 (2.7)	13/487 (2.7)	0.984
80–89 years	4/933 (0.4)	4/446 (0.9)	0/487 (0)	0.052
*Educational level*				
Primary and lower	151/818 (18.5)	47/388 (12.1)	104/430 (24.2)	< 0.001
Middle school	227/818 (27.8)	104/388 (26.8)	123/430 (28.6)	0.566
High school	174/818 (21.3)	89/388 (22.9)	85/430 (19.8)	0.268
College and higher	266/818 (32.5)	148/388 (38.2)	118/430 (17.5)	0.001
*Duration, months*	6.0 (3.0–24.0)	7.5 (3.0–24.0)	6.0 (3.0–24.0)	0.131
*Dry cough*	679/930 (73.0)	338/445 (76.0)	341/485 (70.3)	0.053
*Timing of cough*				
Morning	253/933 (27.1)	124/446 (27.8)	129/487 (26.5)	0.652
Daytime	330/933 (35.4)	173/446 (38.8)	157/487 (32.2)	0.037
Before sleep	254/933 (27.2)	123/446 (27.6)	131/487 (26.9)	0.816
Nighttime	191/933 (20.5)	72/446 (16.1)	119/487 (24.4)	0.002
*Concomotant symptoms*				
Pharyngeal	704/933 (75.5)	325/446 (73.0)	379/487 (77.8)	0.079
Nasal	350/933 (37.5)	160/446 (36.0)	190/487 (39.0)	0.322
Reflux-related	237/933 (25.4)	110/446 (24.7)	127/487 (26.1)	0.620
Respiratory	322/933 (34.5)	151/446 (33.9)	171/487 (35.1)	0.687
*Smoking status*				
Never	764/933 (81.9)	284/446 (63.7)	480/487 (98.6)	< 0.001
Former	55/933 (5.9)	52/446 (11.7)	3/487 (0.6)	< 0.001
Current	114/933 (12.2)	110/446 (24.6)	4/487 (0.8)	< 0.001
*Occupational exposure*	95/933 (10.2)	50/446 (11.2)	45/487 (9.2)	0.320

Data were expressed as n/N (%) and median (interquartile ranges, IQR). The differing denominators used in the calculation of percentages are because of missing data. Pharyngeal symptoms consisted of itchy throat, pharyngeal foreign body sensation, frequent throat clearing, etc. Nasal symptoms comprised runny nose, postnasal dripping, sneezing, etc. Reflux-related symptoms included acid regurgitation, heartburn, belching, etc. Respiratory symptoms included wheezing, dyspnea, chest tightness, etc.

With respect to educational attainment, 18.5 % of the overall patients received primary school or lower education, 27.8 % middle school, 21.3 % high school, 32.5 % college and even higher education.

**Fig. 3:**
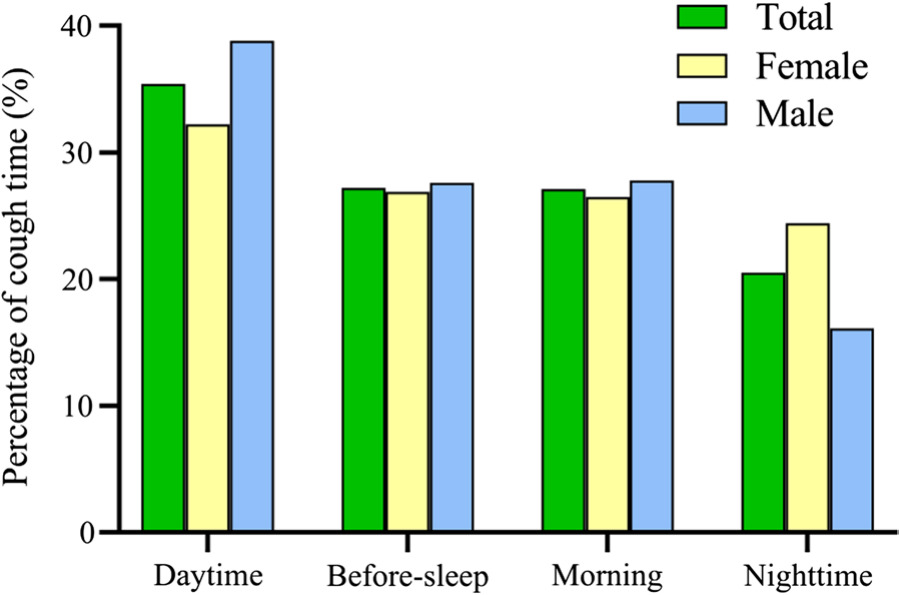
Cough timing distribution in chronic cough patients (n = 933)

The average duration of cough was 6.0 (IQR 3.0–24.0) months, with coughing lasting more than 1 year accounting for 41.5 %. The majority (679, 73.0 %) presented with dry cough. 330 (35.4 %) patients coughed during the daytime, along with before sleep (254, 27.2 %), in the morning (253, 27.1 %) and at night (191, 20.5 %) (Fig. 3). Seasonal cough was reported in 103 (40.7 %) of 253 patients with chronic cough longer than 2 years, especially in winter.

**Fig. 4:**
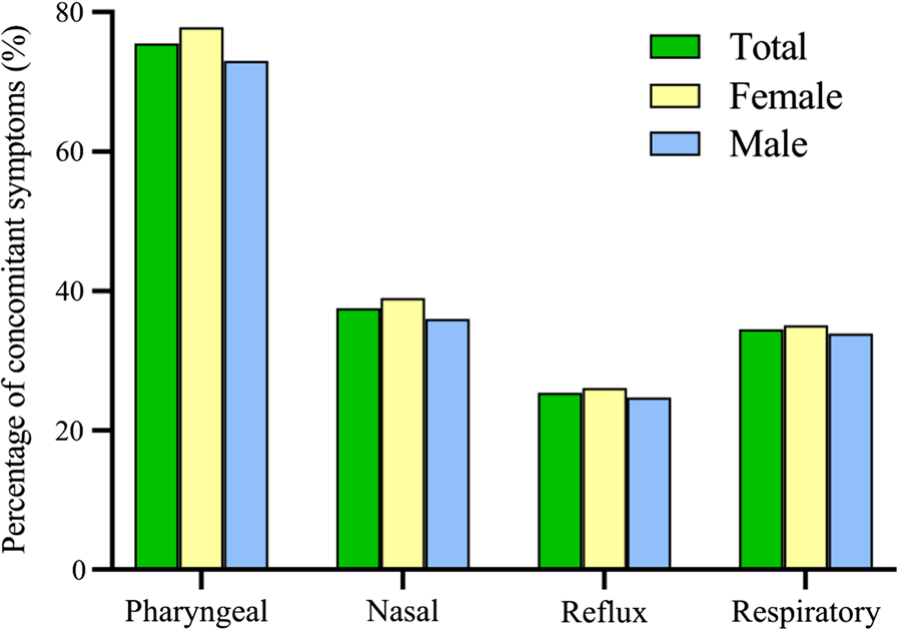
Different concomitant symptoms in chronic cough patients (n = 933). Respiratory symptoms included wheezing, dyspnea, chest tightness, etc.

As depicted in Fig. 4, laryngeal paresthesia was the most common accompanying symptom (704, 75.5 %), followed by rhinitis/sinusitis-related (350, 37.5 %), respiratory (322, 34.5 %) and reflux symptoms (237, 25.4 %). Patients with productive cough exhibited significantly higher incidence of rhinitis/sinusitis-associated symptoms and morning cough compared with those with dry cough (49.0 % vs. 33.0 %, 34.7 % vs. 24.3 %, respectively. both *P* < 0.001).

In terms of smoking history, 81.9 % of patients were lifetime non-smokers, 5.9 % were ex-smokers, and 12.2 % were current smokers. 95 (10.2 %) patients with chronic cough had a history of occupational exposure.

### Characteristics between male and female coughers

As outlined in Table 1, an older age was described in women than men (median [IQR]: 43.0 [33.0–53.0] vs. 37.0 [29.0–50.0] years, *P* < 0.001). In addition, age 50–59 years was more prevalent in females (female vs. male: 20.9 % vs. 12.3 %, *P* < 0.001), but 20–29 years more in males (female vs. male: 13.8 % vs. 24.2 %, *P* < 0.001). Of 818 patients with recording educational attainment, more female patients received primary school and lower education (24.2 % vs. 12.1 %, *P* < 0.001), and more male patients received college and higher education (38.2 % vs. 17.5 %, *P* = 0.001). Nocturnal cough more commonly appeared in women (24.4 % vs. 16.1 %, *P* = 0.002), while cough during the daytime more frequently occurred in men (38.8 % vs. 32.2 %, *P* = 0.037). Most patients (764, 81.9 %) with chronic cough were lifetime never smokers either in males (284, 63.7 %) or in females (480, 98.6 %). It was noteworthy that the overall proportion of ex- and current tobacco users were markedly higher in males than in females (36.3 % vs. 1.4 %, *P* < 0.001).

## Discussion

We evaluated approximately 1000 patients with chronic cough from 13 tertiary hospitals in Guangdong. To our knowledge, this is the first multi-center survey investigating the demographics and clinical characteristics of chronic cough patients in China. Our results demonstrated that, chronic cough patients in Guangdong displayed an equal gender, young profile and high proportion of laryngeal paresthesia, being almost similar to our previous single-center study [8]. In addition, we found that chronic cough females exhibited an older age with a much higher prevalence of nocturnal cough compared with males. Overall, this study provided further evidence of the distinct age and gender distribution of chronic cough patients between China and western countries [7].

The disparities of age and gender distribution in chronic cough population between China and western countries have been reported previously [7, 8]. In 2014, Morice and colleagues found that the majority with chronic cough were old females, being largely uniform across western countries except for China [7]. However, our results showed chronic cough patients displayed a middle-aged predominance and an equal gender distribution, as previously described [8]. Cough reflex hypersensitivity in females, especially post-menopausal women, was thought to be the potential pathophysiologic mechanism of an older female preponderance among the chronic cough patients in western countries [7]. Intriguingly, a recent study by our group suggested Chinese chronic patients shared the similar age and gender difference in cough sensitivity as those patients of western countries [8]. Therefore, a heightened cough reflex sensitivity could not explain the age and sex-related discrepancies between China and western countries. Exposure to cigarette smoking could elicit cough in rodent and human study [14, 15]. A men-to-women ratio of current smokers in our study was 24:1, reflecting the poor correlation between heightened cough reflex sensitivity and tobacco use in females. In the real world, however, some smokers with isolated cough symptom tend to ascribe their cough to tobacco and hence had a low propensity to seek health care to combat it [16], which provided a possible explanation of more women presenting to a physician for the evaluation of chronic cough. Of note, most patients with chronic cough were life-time non-smokers, regardless of males and females. In addition, air pollution level is more serious in China compared to western countries, the annual particulate matter 2.5 concentration was 31–44 ug/m^3^ in the Pearl River Delta Region of China from 2013 to 2017 [17]. Short-term exposure to pollutants could evoke more cough among the patients with chronic obstructive pulmonary disease [18], while a decline of air pollution level was associated with a reduction in prevalence of chronic cough [19]. Furthermore, recent studies mentioned that diverse racial and geographic backgrounds, unhealthy lifestyle, occupational exposure, as well as dietary habit might contribute to these demographic discrepancies of chronic cough patients across different regions [7, 8, 13]. Taken together, it seems that many factors might play a role in the distinct age and gender distribution. Further study needs to clarify it.

Few studies have investigated the impact of educational attainment on the incidence of respiratory conditions. In the present study, up to 67 % of the subjects with chronic cough received a low educational level. A large cohort study with 2,819 subjects over a period of 11 years revealed the incidence of common respiratory symptoms decreased with increasing educational level, such as morning cough, expectoration, chronic cough, dyspnea and wheezing [20]. Adults with low educational attainment had increased vulnerability to occupational exposure and poor health awareness. Moreover, lower educational level might be associated with less health care resource and higher economic burden. These factors would cause their delayed treatment, which turned acute disease status into chronic conditions. To sum up, a correlation might exist between educational attainment and the prevalence of chronic cough. But large epidemiological surveys are needed in the future.

In our study, the median cough duration was 7.0 months, being consistent with those previous reports in general respiratory clinics (average duration 4–12 months) [6, 21, 22]. In clinical practice, some patients frequently complained of their cough for several years [11]. And many patients visiting specialist cough clinics had a longer duration (around 24 months) than those attending the respiratory clinics [8]. A majority of patients with chronic cough would like to firstly consult the physicians of local hospitals in consideration of the limitation of distance, time and expense. However, Morice and colleagues found that, chronic cough patients would like further information to be available on their cough and access to specialist cough clinics, due to the limited efficacy of treatment [23]. In addition, they also observed that 72 % of chronic cough patients had visited their doctors ≥ 3 times in primary and secondary cares, which implied that they had experienced an uncontrolled cough for very long time before attending specialist clinics. The above evidence supported a longer cough duration occurring in patients seeking expert medical opinion. In a word, it was necessary to increase awareness of chronic cough and improve the implementation of cough guidelines in clinical practice, facilitating the early identification of potential causes and effective treatment.

Our findings showed that chronic cough patients typically presented with dry cough, as similar to that in previous studies [8, 24]. Nonetheless, chronic productive cough mostly appeared in patients with chronic bronchitis, bronchiectasis, cystic fibrosis, as well as chronic respiratory infections [25–28]. A prospective study on the systematic assessment of cough characters reported poor added value in determining any specific diagnosis in adults [29]. By contrast, cough characteristics formed a major classification of cough in children and assisted in a diagnosis of protracted bacterial bronchitis (PBB), one of the most common causes of chronic cough in children [30]. We found that patients with productive cough had a higher prevalence of morning cough with concomitant rhinitis/sinusitis-related symptoms compared with those with dry cough, indicating a possible relationship between the production of phlegm and the change of body position. Excessive mucus from the nose were more likely to drop into the throat or lower respiratory tract while asleep. Moreover, productive cough and rhinitis/sinusitis-related symptoms showed well predictive value for diagnosing UACS [9]. Based on the above findings, we inferred that UACS might be one of the most important causes in cases with productive cough.

In the present survey, we observed that cough mostly occurred during the day, which was consistent with the cough measurement of 24-hours automated cough monitors [31]. There was a potential possibility of suppression of the cough reflex by cortical pathways or the reduction of exposure to tussive stimuli during sleep [32–35]. Intriguingly, our results showed women reported more nocturnal cough, being similar to those observations in community population survey both in Australia [36] and Europe [37]. The previous works suggested most females and nocturnal cough in CVA [8, 9], the most common cause of chronic cough in China [6]. Hence, it was reasonable to speculate that CVA might be an important etiology in Cantonese chronic cough population. Recently, studies showed patients with refractory chronic cough were less able to voluntarily suppress capsaicin-evoked cough compared to healthy controls [38, 39]. Whether this impaired ability of cough suppression, especially at night, mostly occurred in female patients with chronic cough remained unclear. Taken together, asthma or allergy, environmental exposure, and decreased cough reflex suppression might play a role in nocturnal cough frequently occurring in females.

The main strengths of our study are chronic cough patients from thirteen tertiary hospitals and unselective data collection, supporting the representativeness of this study. In addition, we also reported the differences of clinical profiles, regardless of in different genders or cough characters. Our study has several limitations. Firstly, this survey was only conducted in respiratory specialist clinics of tertiary hospitals, but not in primary and secondary care centers. Secondly, although the patients were enrolled from 13 sites, the sample is not large. Therefore, our data might not fully reflect demographics and cough features of overall chronic cough population. Lastly, we did not explore the reason why the patients with chronic cough had an equal gender distribution and young profile in this study.

## Conclusions

In summary, our study shows that chronic cough patients displayed an equal gender and young predominance in Guangdong, further highlighting the distinct age and gender distribution between China and Western countries. In addition, female patients present different clinical features from male patients, such as an elder age and more nocturnal cough in women. This implies that further study is needed to elucidate the possible mechanism of these discrepancies, thereby achieving the better understanding and management of chronic cough.

## Data Availability

The datasets used and/or analysed during the current study available from the corresponding author on reasonable request.

## Abbreviations

CVA: Cough variant asthma
UACS: Upper airway cough syndrome
NAEB: Non-asthmatic eosinophilic bronchitis
GERC: Gastroesophageal reflux cough
IQR: Interquartile range
PBB: Protracted bacterial bronchitis
